# *Liza ramada* Juveniles after Exposure to the Toxic Dinoflagellate *Vulcanodinium rugosum*: Effects on Fish Viability, Tissue Contamination and Microalgae Survival after Gut Passage

**DOI:** 10.3390/toxins14060401

**Published:** 2022-06-10

**Authors:** Aurélien Bouquet, Marie Anaïs Perdrau, Mohamed Laabir, Elodie Foucault, Nicolas Chomérat, Jean Luc Rolland, Eric Abadie

**Affiliations:** 1 MARBEC, Université de Montpellier, CNRS, Ifremer, IRD, 87 Avenue Jean Monnet, 34200 Sète, France; perdrauanais@gmail.com (M.A.P.); elodie.foucault@ifremer.fr (E.F.); eric.abadie@ifremer.fr (E.A.); 2 MARBEC, Université de Montpellier, CNRS, Ifremer, IRD, 34095 Montpellier, France; mohamed.laabir@umontpellier.fr; 3 IFREMER, Station de Biologie Marine, Place de la Croix, 29900 Concarneau, France; nicolas.chomerat@ifremer.fr; 4 IFREMER, Biodivenv, 79 Route de Pointe Fort, 97231 Martinique, France

**Keywords:** *Vulcanodinium rugosum*, *Liza ramada*, contamination, pinnatoxins, portimines, food chain, fish, dissemination

## Abstract

Pinnatoxins (PnTX) and Portimines (Prtn), two toxins produced by the benthic dinoflagellate *Vulcanodinium rugosum*, are known to be lethal to mice after intraperitoneal or oral administration. They are also known to accumulate in shellfish such as mussels and clams, but their effect on fish and the upper food chain remains unknown. In this work, juveniles of the fish *Liza ramada* (Mullet) were exposed to a strain of *V. rugosum* producing PnTX G and Prtn A. The fishes’ viability and contamination were recorded at times interval. Results showed that *L. ramada* juveniles were able to feed on *V. rugosum* and that their tissues could be contaminated by PnTX G and Prtn A without impact on fish viability. Furthermore, the microalgae temporary cysts survived and germinated after fish gut passage. This study showed the potential of *L. ramada* to transfer PnTX and Prtn toxins to the upper food chain and to disseminate *V. rugosum* in environment.

## 1. Introduction

Harmful Algal Blooms (HABs) [1] are known to cause significant environmental and health problems, and their frequency and intensity have increased worldwide in recent decades [2,3]. The dissemination of harmful algal species around the world has been shown to be caused by different means: transport by coastal currents, feces of migratory birds [4], transport of water ballast [5,6] or translocation of shellfish stocks [7]. The phycotoxins produced by harmful algae are known to accumulate in tissues of many marine species, including mammals, birds, fish and bivalve mollusks [8]. Hence, these toxins can be transferred to the upper levels of the food chain by predation [8,9,10,11] and become an important threat to human health [12].

*Vulcanodinium rugosum* is a bentho-pelagic toxic dinoflagellate identified by Nezan and Chomerat (2011) and is the producer of two types lipophilic toxins, Pinnatoxins (PnTX) and Portimines (Prtn) [13]. These toxins have been shown to accumulate in bivalve mollusks such as mussels, clams and oysters [14]. Pinnatoxins belong to the Fast Acting Toxins (FAT) and act as acetylcholine competitive antagonists [15], causing neurotoxic effects and death after oral or intraperitoneal administration in mice [16]. Portimines act as an apoptosis inducer and are less toxic to mice at equal doses, but cause death at high concentrations [17]. *V. rugosum* and its toxins have been found in many areas around the world such as China, Japan, New Zealand, Cuba and France [14,18,19,20,21]. In the French Mediterranean Ingril lagoon, record concentrations of PnTX G have been found in mussel tissues (up to 1244 µg PnTX G/kg of whole wet body weight (wbw)) during *V. rugosum* blooming season (June to September) [13,14,15].

Among fish that can be found in French Mediterranean lagoons, the mullet *Liza ramada* is one of the most represented species. Their juveniles are known to have various feeding habits and to feed on many phytoplankton species, benthic and pelagic [22,23,24]. These microphagous omnivores can digest food by crushing sediments in their stomach and guts [25], which makes them sensitive to contaminant absorption [26]. Mullets have been shown to accumulate many contaminants in their tissues, such as heavy metals and herbicides [27,28,29] and toxins including cyanobacteria toxins [30], tetrodotoxins [31] and brevetoxins [32]. Interestingly, the recruitment period of mullets overlaps with the blooming season of *V. rugosum.* In addition, as *V. rugosum*, *L. ramada* juveniles preferential habitat is shallow and near coast water [14,33]. Consequently, they are potential consumers of this harmful alga and a potential reservoir of PnTX and Prtn. As they have many predators such as *Gobius cobitis* (large Gobie) [34] and *Discentrarchus labrax* (Sea bass) [35], juvenile mullets could be a vector for PnTX in the food chain. To our knowledge, no study has investigated the accumulation of PnTX and Prtn or their effects on fish. Moreover, mugilids are regional migrators [36] and juveniles are known for moving within lagoons [37]. Studies showed that harmful algal cysts can survive a passage through marine organisms’ guts, including bivalves mollusk and fish [38,39] and initiate future blooms when released [40,41,42]. To our knowledge, no study has ever investigated the survival of *V. rugosum* after fish gut passage, which could lead to cells dissemination along fish movements.

The aim of this study was to experimentally evaluate the potential of the mullet *Liza ramada* to propagate both *V. rugosum* toxins and cells in the marine environment. By feeding fish with *V. rugosum* at cell concentrations corresponding to the densities observed during natural blooms, we evaluated the toxin’s accumulation in tissues, its effect on animal mortality and the presence and survival of cells in the feces.

## 2. Results

### 2.1. L. ramada Ability to Ingest V. rugosum Cells

*L. ramada* juveniles were fed with various diets: cultures of *V. rugosum* cells, of non-toxic control *Scripsiella acuminata* cells, milled flakes or fast. Apart from the fast, grasping behaviors of the fish could be observed. For all other diets, two kinds of feeding behaviors could be identified: the fish either swallowed particles in the water column or grazed on the bottom of the tanks.

**Figure 1:**
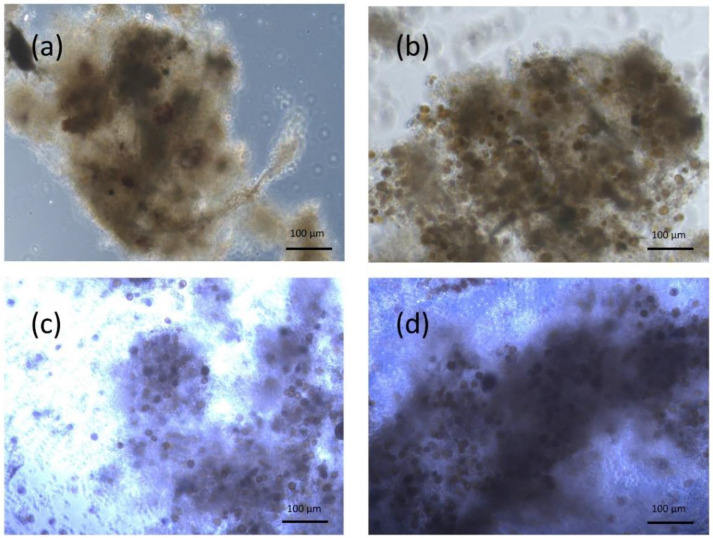
Light microscope photographs of feces produced by *Liza ramada* fed with milled flakes (MF) (**a**), *Scripsiella acuminata* (C0: 120,000 cells/fish/day) (**b**), *Vulcanodinium rugosum* (C1: 32,000 cells/fish/day) (**c**) and *V. rugosum* (C2: 120,000 cells/fish/day) (**d**) after three days of exposure.

Immediately after being fed for three days, several fish were isolated from microalgae exposure and their feces were sampled and observed under light inverted microscopy. No feces were produced by the fasted fish. Feces produced by the fish fed with milled flakes showed no cysts or pelagic microalgal cells (Figure 1). Feces produced by fish fed with *V. rugosum* and *S. acuminata* were composed of a mix of digested cells and intact cells. The intact cells were composed only of temporary cysts (Figure 1).

**Figure 2:**
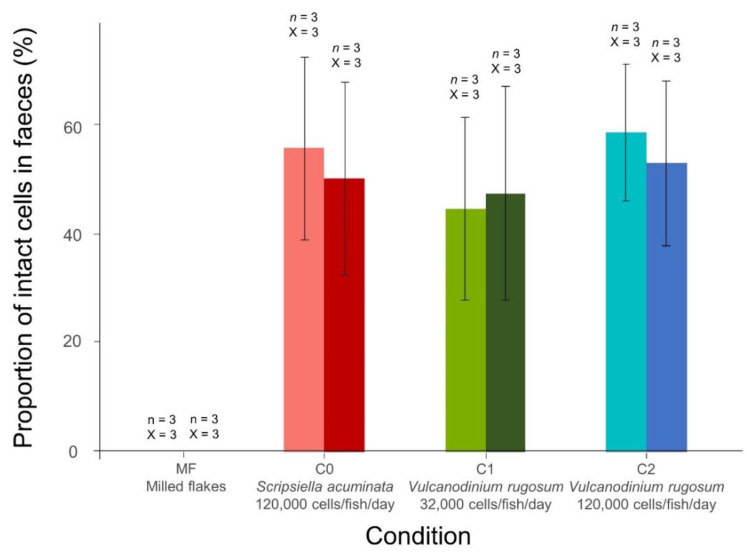
Proportion of intact cells in feces after three days of feeding. In light and dark colors, figure the values on the first and second day of fasting, respectively. *n*: number of fecal ribbons in each replicate. X: number of replicates. The error bars represent ± standard deviation from triplicate mean values.

The first day after isolation from microalgae (from 2 to 10 h after the isolation), the proportion of intact cells within the feces reached 0 ± 0%, 55.6 ± 16.7%, 44.4 ± 16.7% and 58.3 ± 12.5% for the fish fed with milled flakes (MF), *S. acuminata* (C0: 120,000 cells/fish/day), *V. rugosum* (C1: 32,000 cells/fish/day), *V. rugosum* (C2: 120,000 cells/fish/day), respectively (Figure 2). The following day (from 24 to 34 h after the isolation), the proportion reached 0 ± 0%, 50.0 ± 17.7%, 47.2 ± 19.5% and 52.7 ± 15.0% for the MF, C0, C1 and C2 conditions, respectively. No significant difference could be shown between the percentages on day 1 and day 2 (Paired Wilcoxon test *p* value > 0.05) and no significant difference was found between the percentages for the different conditions each day (Kruskal–Wallis test *p* value > 0.05). The fish released a steady proportion of cells in their feces through time, regardless of the microalgae species or quantity they were fed with.

**Figure 3:**
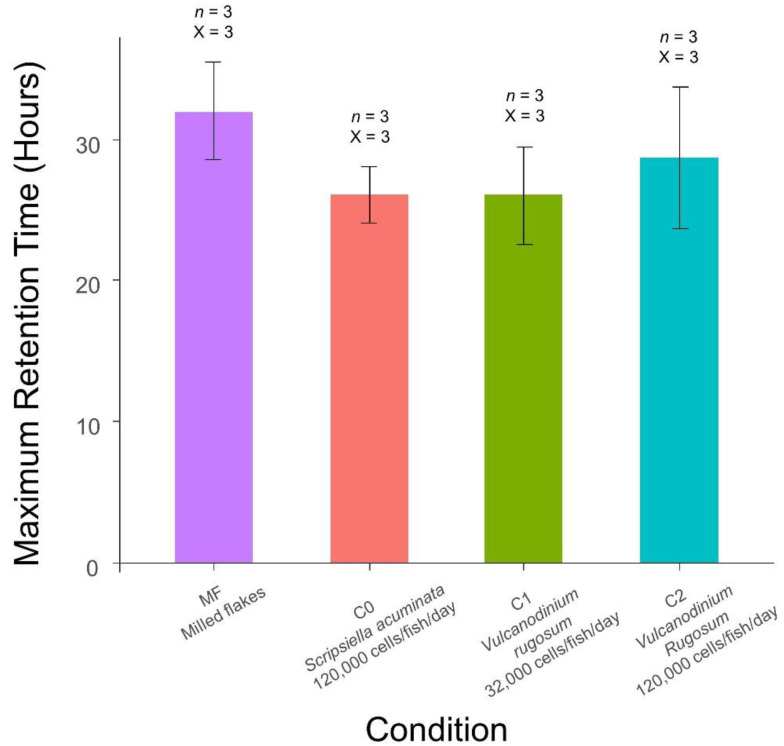
Maximum Retention time after three days of feeding. *n*: number of individuals in each replicate. X: number of replicates. The error bars represent ± standard deviation from triplicate mean values.

The maximum gut retention time was measured from the time fish were isolated from microalgae to the last time feces were produced. It reached 32.0 ± 3.5 h, 26 ± 2 h, 26 ± 3.5 h and 28.7 ± 5.0 for the MF, C0, C1 and C2 conditions, respectively (Figure 3). No impact of the alimentation on the retention time could be shown as the RT max did not differ significantly between all the conditions (Kruskal–Wallis test, *p* value > 0.05). It must be noted that the maximum retention time may in fact be slightly higher as the fish could have stopped eating before they were isolated from microalgae. All together, these results showed that *L. ramada* was able to ingest *V. rugosum* cells without affecting its digestive capability.

### 2.2. V. rugosum Feeding Induced L. ramada Tissues Contamination

**Figure 4:**
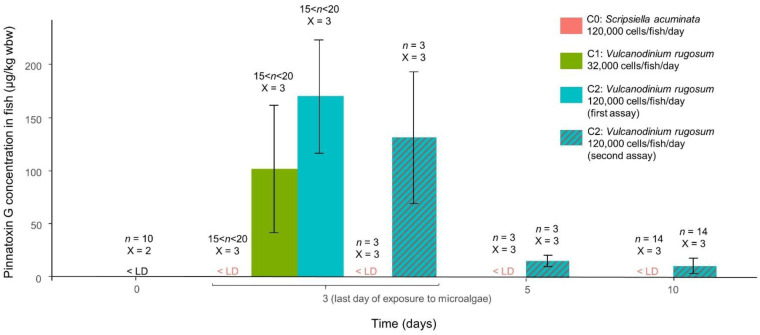
Barplot of mean Pinnatoxin G concentrations found in fish before the experiment (in black), after 3 days of exposure, and 2 days and 7 days after the end of the exposure. *n*: number of individuals per replicate. X: number of replicates. Error bars represent ± standard deviation from triplicate mean values.

Fish exposed to *V. rugosum* and to *S. acuminata* were euthanized for toxin analysis in tissues after 3 days of exposure, and after 2 and 7 days of depuration. *V. rugosum* strain contained 0.83 pg PnTX G/cell and 9.11 pg Prtn A/cell and *S. acuminata* strain had no detectable toxins. No toxins were detected in a representative sample of *Liza ramada* before the beginning of the experiments or in the individuals fed with *S. acuminata* throughout the experiments (Figure 4).

PnTX G was found in fish exposed to 32,000 cells/fish/day at mean concentrations of 101.93 ± 59.96 µg/kg wbw at the 3rd day of the experiment (Figure 4). PnTX G was found in fish exposed to 120,000 cells/fish/day at mean concentrations of 169.82 ± 53.25 and 130.40 ± 61.47 µg/kg wbw for the first and second assay, respectively (Figure 4). No significant difference was shown between the toxins concentrations at 32,000 and 120,000 cells/fish/day (Kruskal–Wallis test, *p* value > 0.05). PnTX G was found at concentration of 15.43 ± 5.18 and 10.99 ± 7.19 µg/kg wbw at the 5th and 10th day of the experiment, respectively. A significant impact of time on PnTX G concentration was revealed through a Friedman test comparing toxin concentrations during the experiment (*p* value = 0.049). Since the fish were exposed to *V. rugosum* up to the 3rd day and digestive tracts of fish were cleared of *V. rugosum* after two days of depuration (see Section 5.2), the drop of concentration between the 3rd and 5th day reveals that most of the toxin found on the 3rd day was from *V. rugosum* cells present in the digestive tubes. However, the post hoc Conover test could not reveal significant differences (*p* value > 0.05 for every pairwise comparisons), which is probably due to the low number of individuals.

**Figure 5:**
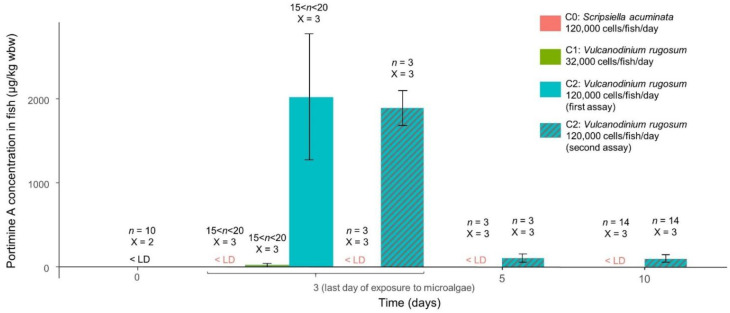
Barplot of mean Portimine A concentrations found in fish before the experiment (in black), after 3 days of exposure, and 2 days and 7 days after the end of the exposure. *n*: number of individuals per replicate. X: number of replicates. Error bars represent ± standard deviation from triplicate mean values.

Prtn A was found in fish exposed to 120,000 cells/fish/day at the 3rd day of the experiment (2025.83 ± 754.17 and 1896.74 ± 207.15 µg/kg wbw for the first and second assay, respectively). Significantly lower concentration was found in fish fed with 32,000 cells/fish/day (2.65 ± 4.58 µg/kg wbw) (Kruskal–Wallis test, *p* value: 0.046) (Figure 5). This result suggested that the contamination with Prtn A was *V. rugosum* dose-dependent. At the 5th and 10th day of the experiment, the concentrations had dropped to 105.53 ± 48.63 and 100.55 ± 45.62 µg/kg wbw, respectively. As for PnTX G, a significant impact of time could be revealed by the Friedman test (*p* value = 0.049), but the post hoc Conover test could not reveal significant differences (Pair wise comparisons: *p* value > 0.05). The experiment revealed a tissue contamination highlighted by the presence of toxins after depuration. The high level of toxin found in fish was coherent with high concentration of intracellular Prtn A found in *V. rugosum* cells.

### 2.3. Lack of V. rugosum Effect on Fish Viability

The viability of *L. ramada* was investigated up to 10 days throughout the feeding experiments. No significant difference in viability rates was found until the end of the experiments between controls and contaminated individuals (Table 1; Kruskal–Wallis test *p* value > 0.05). In addition, any visible effects on fish health were observed for the duration of the experiments: no loss of balance, motility or decrease of the respiratory function or change in physical aspect. This suggested that *V. rugosum* and its toxins have no effect on the viability of *L. ramada*.

### 2.4. V. rugosum Temporary Cysts Germinated after Gut Passage

**Figure 6:**
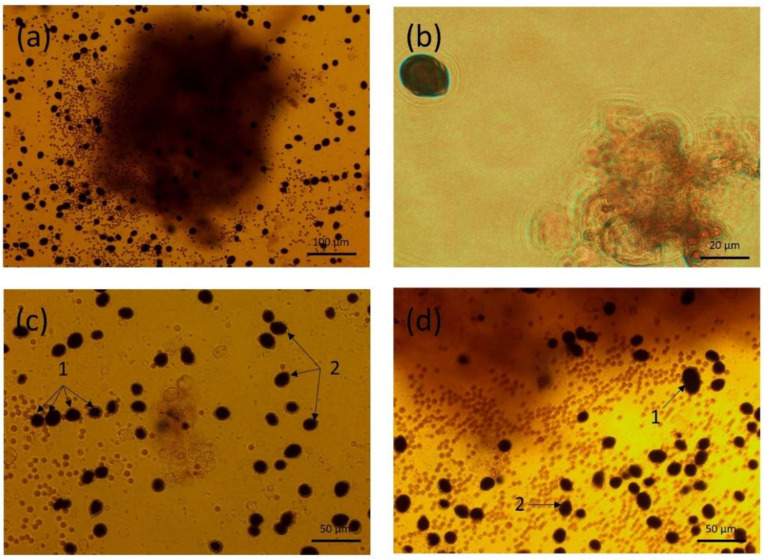
Light microscope photographs of cells in feces produced by *Liza ramada* fed with *Vulcanodinium rugosum* fixed with lugol 24 h after incubation. (**a**) Content of a well. (**b**) A pelagic cell. (**c**,**d**) Mixes of temporary cysts (1) and pelagic cells (2).

After 3 days of exposure to *V. rugosum*, *L. ramada* feces containing *V. rugosum* temporary cysts were dislocated and incubated in a 96-well plate filled with ENSW medium. The feces were then observed every 2 h to check for the presence of pelagic cells. Many pelagic cells were observed after 8 to 22 h of incubation (Figure 6), which showed that temporary cysts had germinated. The minimum time before germination was thus estimated between 8 and 22 h (Table 2). The germination percentage (percentage of temporary cysts that germinated after gut passage) could not be determined because *V. rugosum* cells encysted in wells. The general characteristics of microalgae cells survival after *L. ramada* gut passage are summed up in Table 2.

## 3. Discussion

This study provided the first evidence of the contamination of fish tissues by *V. rugosum* toxins. No effect on fish viability was shown, and the tissue contamination and presence of *V. rugosum* temporary cysts in the fish feces revealed for the first time that mugilids are consumers and potential vectors and disseminators of *V. rugosum.*

### 3.1. Liza ramada Potential to Be a Vector of Vulcanodinium rugosum Toxins

Throughout the feeding experiments, *L. ramada* juveniles swallowed particles in water and grazed on the floor, and temporary cysts were found in fish feces, revealing the ability of *L. ramada* to feed from *V. rugosum* cells. As *V. rugosum* can be found either in water column in its pelagic form, or encysts into temporary cysts and sediment to the bottom [14], this highlighted the capability of *L. ramada* to feed on both *V. rugosum* forms. *V. rugosum* and *S. acuminata* temporary cysts were found in feces in similar quantities, suggesting that the fish were not deterred by *V. rugosum*. This result is in agreement with *L. ramada* natural feeding behavior, which includes many phytoplankton species of various shapes and sizes [22,24]. It suggested that *V. rugosum* is an available food for *L. ramada* in natural habitats.

After 3 days of feeding, and 2 days of depuration allowing gut clearance, toxins were found in *Liza ramada* individuals, which revealed that their tissues were contaminated. PnTX and Prtn are cyclic imines with lipophilic characteristics, and other lipophilic toxins such as Okadaic acid (OA) and Dinophysistoxins (DTX) were shown to contaminate various fish species [43,44,45,46,47]. Prtn A concentration found in *L. ramada* exposed to 120,000 cells/fish/day (105.53 µg/kg wbw) was similar to that of DTX (94.4 µg/kg wbw), or OA (361.1 µg/kg) [43]. In comparison, very low concentration of Prtn A was found in fish exposed to 32,000 cells/fish/day. We hypothesized that this low concentration could be due to toxin metabolization, and that toxin metabolization mechanisms could be saturated when exposed to high levels of toxins. Future studies are needed to investigate these mechanisms. PnTX G concentration (15.43 µg/kg wbw) was lower than that found in the mussel *Mytilus galloprovincialis* (between 70 µg/kg and 80 µg/kg wbw) fed with similar quantity of *V. rugosum* [14]. PnTX G is also a neurotoxin and many studies have shown that other neurotoxins such as Domoic acid and Saxitoxins can contaminate fish tissues [48,49,50,51,52,53]. The presence of toxin in *L. ramada* tissues could be explained by the long gut length of mugilids species, which could increase considerably the quantity of *V. rugosum* cells inside the gut along with the absorption efficiency of the toxins [54,55]. Toxins were still present in similar concentration in tissues 7 days after the exposure, revealing that no detoxification process had occurred. This could be explained by the high lipid contents of fish skin that are effective storage for lipophilic toxins [55,56]. These results showed that *L. ramada* could convey *V. rugosum* toxins in their tissues for at least several days after exposure.

No effect of the neurotoxic dinoflagellate *V. rugosum* was shown on mullet viability. Microalgae concentrations used during the experiments (32,000 and 120,000 cells/fish/day, equivalent to 80 cells/mL/day and 300 cells/mL/day, respectively) were comparable with the highest natural densities of *V. rugosum* observed in bloom events until now (up to 152 cells/mL during summer 2017 in Ingril Lagoon) [57]. Higher microalgae concentrations could have been used, but they would not have been representative of densities observed in natural habitats. *V. rugosum* is known to show important variability in terms of toxin production, based on the environmental conditions and geographical origin [58,59,60]. The strain used here contains high PnTX G concentration (0.81 pg/cell compared to the IFR-VRU-01 strain: 0.14–0.36 pg/cell) [58] and also high Prtn A concentration (9.2 pg/cell compared to the strain from Cienfuegos Bay, Cuba: 0.4 pg/cell) [18]. Under natural conditions, fish eat both zooplankton and phytoplankton species [61]: the experimental conditions could have led to mortality due to impoverished diet, but mugilids are known for their ability to be maintained and studied in captivity [62]. Considering that the high densities used in this experiment did not have an effect on mullet viability even with elevated intracellular toxins concentrations, we assumed that *V. rugosum* would not cause lethal toxicity to mullets in natural habitats. Longer term studies would, however, allow for investigating the impacts of a chronic exposure.

Mugilid juveniles use Mediterranean lagoons as nursery areas [63], and they are widely predated there by *D. labrax* (European Bass) [61,64]. It was also found that large gobies (>29 mm) of the *Pomatoschistus microps* species predate smaller juvenile fish [34] and are thus are inclined to predate *L. ramada* juveniles. It is thus possible that these fish could contaminate themselves by predating *L. ramada*. Maximal levels of toxins in the fish were associated with *V. rugosum* cells’ presence in their guts. As predators eat the whole living prey, maximal risks of mugilid predators’ contamination could occur during and just after *L. ramada* exposure to *V. rugosum*. However, as tissue contamination could be shown, and still remained 7 days after exposure, it is possible that predators could contaminate outside these periods. No studies have yet shown the long-term persistence of PnTX and Prtn in the fish tissues, but some toxins such as ciguatera toxins can persist for several months in the contaminated organisms [36]. The results of this study suggested that *L. ramada* could constitute a vector of *V. rugosum* toxins through trophic chain. Further studies on longer-term persistence could bring more information on this potential throughout their life.

This study does not allow for evaluating if toxins could reach high levels in other fish of the lagoonal food web, but the PnTX G toxins found in *L. ramada* tissues are in the same range as the critical sanitary threshold determined for PnTX G in shellfish (23 µg/kg, no existing data for fish). The accumulation and impacts of PnTX and Prtn on heavily fished predators of lagoonal ecosystems such as *D. labrax* are still unknown and should be investigated through further studies to evaluate sanitary risks.

### 3.2. Liza ramada Potential to Disseminate Vulcanodinium rugosum Cells

The ability of fish species to disperse algae and plants in general depends on a range of key factors such as the alga availability, the gut retention time, the survival after gut passage and the germination rate of the algae [41,65]. These factors were investigated through this study.

The alga availability to *L. ramada* was shown through its ability to eat *V. rugosum* pelagic cells and temporary cysts, highlighted by its feeding behavior and the presence of temporary cysts in the feces. The availability generally depends on the fish feeding behavior and on the size of the algae [42]. *L. ramada* juveniles are known to feed on several plankton species with a wide range of sizes [22], including *V. rugosum* size [13]. In natural conditions, the availability also requires the co-presence of the fish and the algae. *V. rugosum* and *L. ramada* can be found both in shallow water near coast in Mediterranean lagoons [14,37]. *L. ramada* juveniles uses lagoons as a nursery area from January to June [63], whereas the blooms of *V. rugosum* happen from June to September and cysts can potentially be found during the entire year [14]. Thus, this study suggested that *V. rugosum* could be available for *L. ramada* consumption in natural habitats. It would be interesting to confirm in situ this consumption by evaluating the content of *L. ramada* guts in lagoons.

An important survival of *V. rugosum* cells after gut passage was revealed by the presence in high proportions of intact temporary cysts in feces and their germination into a high number of pelagic cells observed after a day of incubation. The survival of cells after gut passage is known to depend on their hardness and the structure of cells walls [66,67]. Some studies have shown that species that produce extracellular mucilage are able to remain viable after passing the fish alimentary tract [68,69,70,71]. *V. rugosum* temporary cysts are able to produce mucilage [14], which could explain the survival of this species. The survival of cysts is known to cause issues when secondary bloom are initiated, as for instance after ingestion by bivalve mollusks followed by their translocation [7,39,41]. This work suggests that the morphology and characteristics of *V. rugosum* temporary cysts allowed them to survive a passage through *L. ramada* gut, which could lead to environmental reseeding.

A gut retention time of 34 h was stated in this study, during which the survival of temporary cysts remained steady. This revealed that *L. ramada* could disseminate *V. rugosum* cells up to 34 h after food uptake. This time did not depend on what the fish were fed with, which was congruent with a previous study revealing no impact of food morphology on fish gut retention time [72]. As it was not possible to determine if the fish ate *V. rugosum* cells until the end of the feeding experiment (from when retention time was recorded), an even longer dispersal time lapse could be observed in the environment. Retention time determines the potential distance among which cells can be dispersed. Mugilids are known to be regional migrators, traveling up to 59 kms per year [36], and juveniles show wide movements within lagoons [37]. All together, these results suggested that mullets could participate in the dispersal of cells from spawning sites along their movements and their migratory routes.

*V. rugosum* temporary cysts were shown to germinate massively within a day after feces production. Studies have shown that ingestion by fish may enhance the probability of germination of plants and the growth of microalgae after returning to water, which could be explained by nutrient uptakes during gut passage [71,73,74,75]. It appeared complex to determine the germination rate of *V. rugosum* because of its ability to encyst in laboratory conditions. However, the extremely high number of pelagic cells observed after 1 day of incubation suggested that temporary cysts released by *L. ramada* could contribute to environmental contamination.

## 4. Conclusions

This study showed that *L. ramada* juveniles were able to ingest both pelagic and benthic forms of *V. rugosum*, which had no impact on fish viability but induced a tissue contamination by PnTX G and Prtn A. This work also highlighted the survival of *V. rugosum* temporary cysts and their capacity to germinate after gut passage. Taken together, the results suggested that *L. ramada* could constitute a vector of toxins through the upper trophic chain and disperse *V. rugosum* cells along their movements, increasing thus the impact of this toxic dinoflagellate on the environment. Studies have reported that toxin accumulation in marine organisms can lead to conversion to derivatives showing wide ranges of toxicity [76,77]. Further studies focusing on time series changes in the composition, chemical structure and toxicity of PnTX and Prtn during accumulation in fish would provide critical information on the risks they represent. It would also be interesting to expand experiments to in situ studies in order to ensure *V. rugosum* availability and the risk of *L. ramada* contamination in its natural habitats. Moreover, other phytoplankton feeders in Mediterranean lagoons, such as Atherinidae species or the migratory eel *Anguilla anguilla* [78,79] known to share this primary consumer trophic level [80,81], should be investigated in order to bring more information on environmental risks.

## 5. Materials and Methods

### 5.1. Dinoflagellate Culture

**Figure 7:**
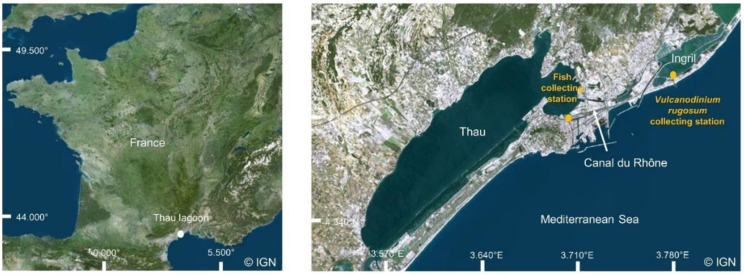
Fish and *Vulcanodinium rugosum* collecting station in Thau and Ingril lagoons.

The *V. rugosum* strain used in this study was isolated from Ingril lagoon (Figure 7) in 2013 and named Ingril 5.48. This strain produces PnTX G and Prtn A. It is cultivated in Enriched Natural Sea Water (ENSW), composed of Thau lagoon water (kept at obscurity for several months, filtered at 0.2 μm, and autoclaved) enriched with sodium nitrate, Ferric EDTA, monosodium phosphate, vitamins and other oligo-elements [82]. The cultures were maintained in batch mode in 50 mL and 250 mL Nunc^TM^ non-treated culture flasks with a photon flux density of 100 μmole·m^−2^·s^−1^ and a photoperiod of 12 h/12 h, a salinity of 35 and a temperature of 25 °C. Frequent inoculations of new culture medium were performed to ensure the survival of the cultured strains.

The control microalgae *S. acuminata* is a non-toxic dinoflagellate similar to *V. rugosum* in size (20–40 µm), shape and life cycle with a pelagic and a benthic stage [83]. The strain, isolated from the South China Sea, was cultivated under the same conditions as *V. rugosum* except for the temperature, which was kept at 20 °C.

### 5.2. Collection and Maintenance of Juvenile Fish Samples

*L. ramada* juveniles (mean total length TL ± SE: 2.64 ± 0.38 cm) were sampled in a time-lapse of 10 days in March (recruitment period from January to June) [63]. The fish were collected in tributary canals of Thau Lagoon connected to the Mediterranean Sea (43°23′48″ N 3°39′38″ E) (Figure 7), using landing nets of 2 mm mesh. Individuals were captured in Thau lagoon to reduce the risk of initial contamination, given the high abundances of *V. rugosum* in Ingril lagoon. Thau and Ingril lagoons are connected with the Canal du Rhône. During the sampling period, and for the acclimation phase, the fish were randomly placed in two 60 L glass aquaria, filled with Thau lagoon water of which 30% was changed every week. Water was filtered using two aquarium filters (AquaFlow 50 SuperFish, Aquadistri BV, Klundert, The Netherlands). They were acclimatized for at least 3 weeks. The physio-chemical parameters were as follows: a constant temperature of 20 ± 1 °C, a salinity of 38 ± 1, a pH of 7 ± 1 units, constant oxygenation, constant filtration and photoperiod (day/night) of 16 h/8 h. During this period, they were fed every two days with milled flakes (TetraMin Flakes, Bio Active, Tetra, Melle, Germany).

### 5.3. Feeding Experiments

**Figure 8:**
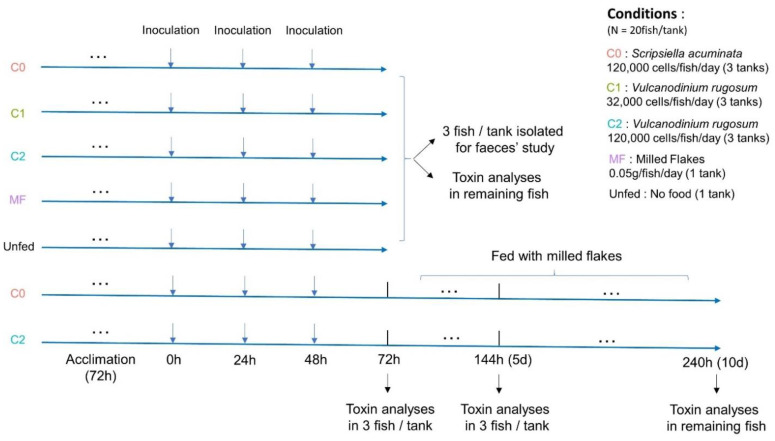
Diagram of the feeding experiments.

Juveniles of *L. ramada* were randomly selected and transferred from the acclimatization aquaria to new aquaria containing 8 liters of filtered (0.7 µm) seawater (20 individuals per aquarium). Fish were fed with *V. rugosum* at concentrations close to 32,000 cells/fish/day (or 80 cells/mL/day) (condition named C1, three aquaria) or 120,000 cells/fish/day (or 300 cells/mL/day) (C2, three aquaria) corresponding to in situ blooms in Mediterranean lagoons [57], or with the non-toxic microalga *S. acuminata* at concentration close to 120,000 cells/fish/day (or 300 cells/mL/day) (C0, three aquaria), or with milled flakes (MF, 0.05 g/fish/day, one aquarium) or unfed (one aquarium). Over the course of three days, fish were fed once a day by adding the amount of food needed. Physical and chemical parameters were controlled every 24 h and were maintained at a temperature of 20.5 ± 0.8 °C, salinity of 37.7 ± 0.6, pH of 8.02 ± 0.17, oxygen content of 92.1 ± 4.8% and NO_2_ + NO_3_ concentration of 9.65 ± 6.48 µmol·L. As cells of *V. rugosum* can encyst and glue to the tank walls, it was impossible to evaluate the ingestion rate of the fish by measuring cells abundances in tanks. To estimate the algae ingestion by *L. ramada*, fish grasping behavior was observed throughout the experiment and the composition of fecal ribbons was analyzed using a photonic microscope. Then, 72 h after the beginning of the experiment, three fish per tank were sampled for the study of *V. rugosum* cells viability after gut passage. The other surviving fish were euthanized and toxins were analyzed in their tissues. In order to evaluate the impact of *V. rugosum* on a longer term, this experiment was renewed with only the C0 and C2 conditions maintained for 72 h: afterward, water was changed and fish were fed with milled flakes and were randomly sampled and euthanized at the 5th and 10th days for toxin analysis. The feeding experiments are described in Figure 8.

### 5.4. Fish Mortality Monitoring

Throughout the feeding experiments, the juveniles’ mortality was monitored every day up to ten days. Each day, potential visible effects of toxins on fish health were checked, including sign of disease or physiological and attitude-wise stress indicator, such as loss of reactivity induced by stimulation, decreased motility, change in feeding behavior, or change of color. All dead fish were to be removed a maximum of 12 h after death.

### 5.5. Viability of Microalgae Cells after Gut Passage

#### 5.5.1. Fecal Material Composition

After three days of feeding experiment, three fish per tank were isolated in separate 2 L filtered (0.7 µm) seawater aquaria. They were fasted for three days and kept under close observation all through the day. Their feces were collected just after being produced using a Pasteur glass pipette and examined under optical Olympus IMT2 (Tokyo, Japan) inverted light microscope within 30 mn. The relative abundance of digested brown bulk material and intact and visible cells in the feces was estimated and feces were photographed by digital camera. At least three feces per aquarium were examined every day.

#### 5.5.2. Germination of Microalgae Temporary Cysts

After optical examination, feces were dislocated and put in culture plates (96 wells) with 200 µL of ENSW. Potential germination was checked every two hours under optical Olympus IMT2 inverted light microscope. The germination rate (in hours) was estimated by the time of first observation of pelagic cells. The germination percentage (percentage of temporary cysts that germinated after gut passage) could not be determined as *V. rugosum* cells encyst naturally in laboratory conditions, making it impossible to distinguish the cells that went through guts as cysts from those that encysted during the incubation.

### 5.6. Toxins Analyses

#### 5.6.1. Extraction from Microalgae Strains

Methanol extractions of the toxin contents from *V. rugosum* and *S. acuminata* strains were performed. An aliquot of 5 mL was taken from one of the culture flasks and fixed using Lugol’s iodine solution. The exact cell concentrations for each strain were then determined using a Nageotte counting chamber via an Olympus IMT2 inverted light microscope.

Secondly, toxin extractions were performed according to Abadie’s protocol [59]. Exactly 20 mL of the culture suspensions was sampled from the flasks and centrifuged (2000× *g*, 15 min, 20 °C). The supernatants were then carefully removed and discarded. 1 ml of 100% methanol (VWR chemicals, Radnor, PA, USA) was then added to the remaining pellets. The extraction was performed by two consecutive sonications of 1 min each, followed by filtration over a 0.2 µm membrane (Whatman Mini-UniPrepTM). The filtered extracts were then stored at −21 °C until quantification.

#### 5.6.2. Extraction from the Fish

All fish sampled for toxins analysis were euthanized (overdose of TMS (Tricaine methanesulfonate) (MS222 140 mg·L)), measured (mean ± sd size (length: head to tail) of 2.62 ± 0.40 cm) and weighed (mean ± sd weigh of 0.14 ± 0.036 g). All samples from the same replicate were pooled and kept at −21 °C until toxin extraction. For each replicate of each condition, the surviving individuals were pooled. The extraction was undergone according to Hess protocol [60]. Toxins were extracted in pooled fish (between 2 and 3 g of tissues) in methanol (9 mL) using a high-speed homogenizer (Ultra Turrax, IKA-werke, Staufen im Breisgau, Germany) at 15,000 rpm for 2 min, and then centrifuged at 3700× *g* for 10 min (Sigma 3-18K, Sigma GmbH, Osterode am Harz, Germany). This extraction was repeated twice and supernatants were transferred to volumetric flasks, which were filled up to 20 mL with MeOH. Aliquots of extracts were filtered (0.2 µm membrane filters) and stored at −21 °C until toxin analyses. For dead individuals at the end of the experiment, the same protocol was followed, but due to the low number of individuals and hence the low tissue weight, the methanol volume used was decreased to 1.5 mL.

#### 5.6.3. Identification and Quantification of Toxins

Quantification of PnTX G and Prtn A were carried out according to Hess protocol [60] using liquid chromatography coupled to tandem mass spectrometry (LC-MS/MS, UFLC XR, Shimadzu, Kyoto, Japan). Certified standard solution of PnTX G (1.92 ± 0.09 µmol·L) and a solution of Prtn A (purified, non-certified) were obtained from the National Research Council in Halifax, Canada. Chromatographic separation was achieved on a Phenomenex Kinetex C18 (100 × 2.1 mm, 2.6 μm) column at 25 °C for analysis (injection volume of 5 μL). The analysis was conducted at a flow rate of 0.8 mL·min.

### 5.7. Data Treatment and Statistical Analysis

All data analyses were performed using R software (version 4.0.2, Vienna, Austria, 2020) [84]. For the contamination experiments, mean concentrations of PnTX G and Prtn A in the fish fed with 32,000 cells and 120,000 cells/fish/day at the 3rd day were compared using the test of Kruskal–Wallis. The impact of time on toxins concentrations in the ten days follow up was analyzed using a Friedman test. If significant, this test was followed by a post hoc pairwise comparison using the Conover test. Non-detectable concentrations were not included in the analysis. For the study of feces, the proportion of intact cells between the three conditions for each time and the maximum gut retention time were analyzed using a Kruskal–Wallis test. The proportion of intact cells between day one and day two were analyzed using a Paired Wilcoxon test.

## Figures and Tables

**Table 1:** Number of deaths for each aquarium during the feeding experiments.

Experiment			Days				
Aquarium (20 Individuals per Aquarium at t0)	Food	Quantity of Food	1	2	3	5	10
			Number of Deaths				
Unfed	-	-	0	0	0	-	-
Milled flakes	Milled Flakes	0.05 g/fish/day	0	0	0	-	-
C0.1	*Scripsiella acuminata*	120,000 cells/fish/day	0	0	0	-	-
C0.2			0	0	1	-	-
C0.3			0	0	0	-	-
C0.4			0	0	0	0	0
C0.5			0	0	0	0	0
C0.6			0	0	0	0	0
C1.1	*Vulcanodinium rugosum*	32,000 cells/fish/day	0	0	2	-	-
C1.2			0	0	1	-	-
C1.3			0	0	0	-	-
C2.1	*Vulcanodinium rugosum*	120,000 cells/fish/day	0	0	0	-	-
C2.2			0	0	1	-	-
C2.3			0	0	0	-	-
C2.4			0	0	0	0	0
C2.5			0	0	0	0	0
C2.6			0	0	0	0	0

**Table 2:** *Sripsiella acuminata* and *Vulcanodinium rugosum* characteristics for survival after *Liza ramada* gut passage. RT_max_: the maximum cells retention time (in hours), Proportion of intact cells: the proportion of intact visible cells in feces in regard to the brown bulk material (in %), Time before germination: the time before germination (in hours) of fish-ingested cells after gut passage. Each mean is calculated on 3 replicates of 3 fish.

Microalga Species	Cells Concentration in Aquaria (Cells/Fish/Day)	RT_max_ (h)	Proportion of Intact Cells (%)		Time before Germination (h)
			2 to 10 h	24 to 34 h	
*Scripsiella acuminata*	120,000	32 ± 3.5	55.6 ± 16.7	50.0 ± 17.7	-
*Vulcanodinium rugosum*	32,000	26 ± 2	44.4 ± 16.7	47.2 ± 19.5	8 to 22
	120,000	26 ± 3.5	58.3 ± 12.5	52.8 ± 15.0	8 to 22

## Data Availability

Not applicable.

## References

[1] Smayda T.J. (1997). Harmful Algal Blooms: Their Ecophysiology and General Relevance to Phytoplankton Blooms in the Sea. Limnol. Oceanogr..

[2] Glibert P.M. (2020). Harmful algae at the complex nexus of eutrophication and climate change. Harmful Algae.

[3] Gobler C.J. (2020). Climate Change and Harmful Algal Blooms: Insights and perspective. Harmful Algae.

[4] Nehring S. (1998). Non-indigenous phytoplankton species in the North Sea: Supposed region of origin and possible transport vector. Arch. Fish. Mar. Res..

[5] Bolch C.J.S., de Salas M.F. (2007). A review of the molecular evidence for ballast water introduction of the toxic dinoflagellates *Gymnodinium catenatum* and the *Alexandrium tamarensis* complex to Australasia. Harmful Algae.

[6] Garrett M.J., Puchulutegui C., Selwood A.I., Wolny J.L. (2014). Identification of the harmful dinoflagellate *Vulcanodinium rugosum* recovered from a ballast tank of a globally traveled ship in Port Tampa Bay, Florida, USA. Harmful Algae.

[7] Scarratt A.M., Scarratt D.J., Scarratt M.G. (1993). Survival of live *Alexandrium tamarense* cells in mussel and scallop spat under simulated transfer conditions. J. Shellfish Res..

[8] Shumway S.E., Allen S.M., Dee Boersma P. (2003). Marine birds and harmful algal blooms: Sporadic victims or under-reported events?. Harmful Algae.

[9] Alves T., Mafra L. (2018). Diel Variations in Cell Abundance and Trophic Transfer of Diarrheic Toxins during a Massive Dinophysis Bloom in Southern Brazil. Toxins.

[10] Choi M.-C., Yu P.K.N., Hsieh D.P.H., Lam P.K.S. (2006). Trophic transfer of paralytic shellfish toxins from clams (*Ruditapes philippinarum*) to gastropods (*Nassarius festivus*). Chemosphere.

[11] Karjalainen M., Reinikainen M., Spoof L., Meriluoto J.A.O., Sivonen K., Viitasalo M. (2005). Trophic transfer of cyanobacterial toxins from zooplankton to planktivores: Consequences for pike larvae and mysid shrimps. Environ. Toxicol..

[12] Botana L.M., Alfonso A. (2015). Phycotoxins: Chemistry and Biochemistry.

[13] Nézan E., Chomérat N. (2011). *Vulcanodinium rugosum* gen. et sp. nov. (Dinophyceae), un Nouveau Dinoflagellé Marin de la Côte Méditerranéenne Française. Cryptogam. Algol..

[14] Abadie E. (2015). Etude de Vulcanodinium rugosum (Dinoflagellé Producteur de Pinnatoxines) se Développant Dans la Lagune Méditerranéenne de l’Ingril. Doctoral Dissertation.

[15] Delcourt N., Lagrange E., Abadie E., Labadie M., Sinno-Tellier S., Bloch J., Arnich N., Molgó J., de Haro L., Mattei C. (2021). Pinnatoxines: Évaluation des risques et toxicovigilance. Toxicol. Anal. Clin..

[16] Munday R., Selwood A.I., Rhodes L. (2012). Acute toxicity of pinnatoxins E, F and G to mice. Toxicon.

[17] Selwood A.I., Wilkins A.L., Munday R., Shi F., Rhodes L.L., Holland P.T. (2013). Portimine: A bioactive metabolite from the benthic dinoflagellate *Vulcanodinium Rugosum*. Tetrahedron Lett..

[18] Moreira-González A.R., Comas-González A., Valle-Pombrol A., Seisdedo-Losa M., Hernández-Leyva O., Fernandes L.F., Chomérat N., Bilien G., Hervé F., Rovillon G.A. (2021). Summer bloom of *Vulcanodinium rugosum* in Cienfuegos Bay (Cuba) associated to dermatitis in swimmers. Sci. Total Environ..

[19] Rhodes L., Smith K., Selwood A., Mcnabb P., Munday R., Suda S., Molenaar S., Hallegraeff G. (2011). Dinoflagellate *Vulcanodinium rugosum* identified as the causative organism of pinnatoxins in Australia, New Zealand and Japan. Phycologia.

[20] Selwood A.I., Wilkins A.L., Munday R., Gu H.F., Smith K.F., Rhodes L.L., Rise F. (2014). Pinnatoxin H: A new pinnatoxin analogue from a South China Sea *Vulcanodinium rugosum* isolate. Tetrahedron Lett..

[21] Smith K.F., Rhodes L.L., Suda S., Selwood A.I. (2011). A dinoflagellate producer of pinnatoxin G, isolated from sub-tropical Japanese waters. Harmful Algae.

[22] Almeida P.R. (2003). Feeding ecology of *Liza ramada* (Risso, 1810) (Pisces, Mugilidae) in a south-western estuary of Portugal. Estuar. Coast. Shelf Sci..

[23] Laffaille P., Feunteun E., Lefebvre C., Radureau A., Sagan G., Lefeuvre J.C. (2002). Can Thin-lipped Mullet Directly Exploit the Primary and Detritic Production of European Macrotidal Salt Marshes?. Estuar. Coast. Shelf Sci..

[24] Lebreton B., Richard P., Parlier E.P., Guillou G., Blanchard G.F. (2011). Trophic ecology of mullets during their spring migration in a European saltmarsh: A stable isotope study. Estuar. Coast. Shelf Sci..

[25] Cardona L., Crosetti D., Blaber S.J.M. (2015). Food and Feeding of Mugilidae. Biology, Ecology and Culture of Grey Mullets (Mugilidae).

[26] Stewart I., Eaglesham G.J., McGregor G.B., Chong R., Seawright A.A., Wickramasinghe W.A., Sadler R., Hunt L., Graham G. (2012). First Report of a Toxic *Nodularia spumigena* (Nostocales/Cyanobacteria) Bloom in Sub-Tropical Australia. II. Bioaccumulation of Nodularin in Isolated Populations of Mullet (*Mugilidae*). Int. J. Environ. Res. Public. Health.

[27] Biagianti-Risbourg S., Bastide J. (1995). Hepatic perturbations induced by a herbicide (atrazine) in juvenile grey mullet *Liza ramada* (Mugilidae, Teleostei): An ultrastructural study. Aquat. Toxicol..

[28] Chouba L., Kraiem M., Njimi W., Tissaoui C.H., Thompson J.R., Flower R.J. (2007). Seasonal variation of heavy metals (Cd, Pb and Hg) in sediments and in mullet, *Mugil cephalus* (Mugilidae), from the Ghar El Melh Lagoon (Tunisia). Transit. Waters Bull..

[29] Taylor D., Maddock B.G., Mance G. (1985). The acute toxicity of nine ‘grey list’ metals (arsenic, boron, chromium, copper, lead, nickel, tin, vanadium and zinc) to two marine fish species: Dab (*Limanda limanda*) and grey mullet (*Chelon labrosus*). Aquat. Toxicol..

[30] Vasconcelos V. (2001). Cyanobacteria toxins: Diversity and ecological effects. Limnetica.

[31] Ben-Gigirey B., Rossignoli A.E., Riobó P., Rodríguez F. (2020). First Report of Paralytic Shellfish Toxins in Marine Invertebrates and Fish in Spain. Toxins.

[32] Woofter R.T., Brendtro K., Ramsdell J.S. (2005). Uptake and Elimination of Brevetoxin in Blood of Striped Mullet (*Mugil cephalus*) after Aqueous Exposure to *Karenia brevis*. Environ. Health Perspect..

[33] Rochard E., Elie P. (1994). La macrofaune aquatique de l’estuaire de la Gironde: Contribution au livre blanc de l’Agence de l’eau Adour Garonne. Etat des Connaissances sur l’estuaire de la Gironde.

[34] Pasquaud S., Girardin M., Elie P. (2014). Diet of gobies of the genus *Pomatoschistus* (*P. microps* and *P. minutus*), in the Gironde estuary (France). Cybium Int. J. Ichthyol..

[35] Barnabé G. (1980). Exposé Synoptique des Données Biologiques sur le Loup ou Bar: Dicentrarchus Labrax (Linné, 1758).

[36] Lassus P., Marcaillou-Le Baut C., Frémy J.-M., Lassus P. (2001). Contamination, Transformation et détoxification des Produits Marins, in Toxines D’algues Dans L’alimentation.

[37] Bourquard C., Benharrat K. (1985). La colonisation des lagunes du Golfe du Lion par les stades jeunes de Soleidae, Mugilidae et Sparidae, 1985. Actes du 110e Congrès National des Sociétés Savantes.

[38] Bauder A.G., Cembella A.D. (2000). Viability of the toxic dinoflagellate *Prorocentrum lima* following ingestion and gut passage in the bay scallop *Argopecten irradians*. J. Shellfish Res..

[39] Guéguen M., Lassus P., Laabir M., Bardouil M., Baron R., Séchet V., Truquet P., Amzil Z., Barillé L. (2008). Gut passage times in two bivalve molluscs fed toxic microalgae: *Alexandrium minutum*, *A. catenella* and *Pseudo-nitzschia calliantha*. Aquat. Living Resour..

[40] Carriker M.R., Carlton J.T., Rosenfield A. (1994). Introductions and transfers of molluscs: Risk consideration and implications. Molluscan Introductions and Transfers.

[41] Laabir M., Gentien P. (1999). Survival of toxic dinoflagellates after gut passage in the Pacific oyster *Crassostrea gigas* Thunburg. J. Shellfish Res..

[42] Görgényi J., Boros G., Vitál Z., Mozsár A., Várbíró G., Vasas G., Borics G. (2016). The role of filter-feeding Asian carps in algal dispersion. Hydrobiologia.

[43] Corriere M., Baptista M., Paula J.R., Repolho T., Rosa R., Reis Costa P., Soliño L. (2020). Impaired fish swimming performance following dietary exposure to the marine phycotoxin okadaic acid. Toxicon.

[44] Souid G., Souayed N., Haouas Z., Maaroufi K. (2018). Does the phycotoxin Okadaic acid cause oxidative stress damages and histological alterations to seabream (*Sparus aurata*)?. Toxicon.

[45] Neves R.A.F., Nascimento S.M., Santos L.N. (2020). Sublethal fish responses to short-term food chain transfer of DSP toxins: The role of somatic condition. J. Exp. Mar. Biol. Ecol..

[46] Ajuzie C.C. (2008). Toxic *Prorocentrum lima* induces abnormal behaviour in juvenile sea bass. J. Appl. Phycol..

[47] Corriere M., Soliño L., Costa P.R. (2021). Effects of the Marine Biotoxins Okadaic Acid and Dinophysistoxins on Fish. J. Mar. Sci. Eng..

[48] Nogueira I., Lobo-da-Cunha A., Afonso A., Rivera S., Azevedo J., Monteiro R., Cervantes R., Gago-Martinez A., Vasconcelos V. (2010). Toxic Effects of Domoic Acid in the Seabream *Sparus aurata*. Mar. Drugs.

[49] Lefebvre K.A., Dovel S.L., Silver M.W. (2001). Tissue distribution and neurotoxic effects of domoic acid in a prominent vector species, the northern anchovy *Engraulis mordax*. Mar. Biol..

[50] Hardy R.W., Scott T.M., Hatfield C.L., Barnett H.J., Gauglitz E.J., Wekell J.C., Eklund M.W. (1995). Domoic acid in rainbow trout (*Oncorhynchus mykiss*) feeds. Aquaculture.

[51] Bakke M.J., Hustoft H.K., Horsberg T.E. (2010). Subclinical effects of saxitoxin and domoic acid on aggressive behaviour and monoaminergic turnover in rainbow trout (*Oncorhynchus mykiss*). Aquat. Toxicol..

[52] Silva de Assis H.C., da Silva C.A., Oba E.T., Pamplona J.H., Mela M., Doria H.B., Guiloski I.C., Ramsdorf W., Cestari M.M. (2013). Hematologic and hepatic responses of the freshwater fish *Hoplias malabaricus* after saxitoxin exposure. Toxicon.

[53] Lefebvre K., Silver M., Coale S.L., Tjeerdema R. (2002). Domoic acid in planktivorous fish in relation to toxic *Pseudo-nitzschia* cell densities. Mar. Biol..

[54] We O. (1968). Utilization of the Direct Grazing and Plant Detritus Food Chains by the Striped Mullet *Mugil cephalus*. https://agris.fao.org/agris-search/search.do?recordID=XF2016004266.

[55] Waltham N.J., Teasdale P.R., Connolly R.M. (2013). Use of flathead mullet (*Mugil cephalus*) in coastal biomonitor studies: Review and recommendations for future studies. Mar. Pollut. Bull..

[56] Lewis R. (2006). Fish cutaneous mucus: A new source of skin surface lipid. Lipids.

[57] (2021). REPHY dataset—French Observation and Monitoring program for Phytoplankton and Hydrology in coastal waters. Metropolitan Data.

[58] Abadie E., Muguet A., Berteaux T., Chomérat N., Hess P., Roque D’OrbCastel E., Masseret E., Laabir M. (2016). Toxin and Growth Responses of the Neurotoxic Dinoflagellate *Vulcanodinium rugosum* to Varying Temperature and Salinity. Toxins.

[59] Abadie E., Kaci L., Berteaux T., Hess P., Sechet V., Masseret E., Rolland J., Laabir M. (2015). Effect of Nitrate, Ammonium and Urea on Growth and Pinnatoxin G Production of *Vulcanodinium rugosum*. Mar. Drugs.

[60] Philipp H., Abadie E., Hervé F., Berteaux T., Séchet V., Aráoz R., Molgó J., Zakarian A., Sibat M., Rundberget T. (2013). Pinnatoxin G is responsible for atypical toxicity in mussels (*Mytilus galloprovincialis*) and clams (*Venerupis decussata*) from Ingril, a French Mediterranean lagoon. Toxicon.

[61] Hichem Kara M., Quignard J.P. (2018). Les Poissons des Lagunes et des Estuaires de Méditerranée.

[62] Ortiz-Zarragoitia M., Bizarro C., Rojo-Bartolomé I., de Cerio O., Cajaraville M., Cancio I. (2014). Mugilid Fish Are Sentinels of Exposure to Endocrine Disrupting Compounds in Coastal and Estuarine Environments. Mar. Drugs.

[63] Cambrony M. (1984). Identification et périodicité du recrutement des juvéniles de Mugilidae dans les littoraux du Languedoc-Roussillon. Vie MilieuLife Environ..

[64] Faucher K., Lagardère J.-P., Aubert A. (2005). Quantitative Aspects of the Spatial Distribution and Morphological Characteristics of the Sea Bass (*Dicentrarchus labrax* L.; Teleostei, Serranidae) Trunk Lateral Line Neuromasts. Brain. Behav. Evol..

[65] Pollux B.J.A. (2011). The experimental study of seed dispersal by fish (ichthyochory). Freshw. Biol..

[66] Moriarty D.J.W. (1973). The physiology of digestion of blue-green algae in the cichlid fish. Tilapia Nilotica J. Zool..

[67] Moriarty C.M., Moriarty D.J.W. (1973). Quantitative estimation of the daily ingestion of phytoplankton by *Tilapia nilotica* and *Haplochromis nigripinnis* in Lake George, Uganda. J. Zool..

[68] Miura T., Wang J. (1985). Chlorophyll a found in feces of phytoplanktivorous cyprinids and its photosynthetic activity. SIL Proc..

[69] Vörös L., Oldal I., Présing M., Balogh K.V., Kufel L.L., Prejs A., Rybak J.I. (1997). Size-Selective Filtration and Taxon-Specific Digestion of Plankton Algae by Silver Carp (Hypophthalmichthys molitrix Val.), in Shallow Lakes ’95: Trophic Cascades in Shallow Freshwater and Brackish Lakes.

[70] Datta (Saha) S., Jana B.B. (1998). Control of bloom in a tropical lake: Grazing efficiency of some herbivorous fishes. J. Fish Biol..

[71] Lewin W.-C., Kamjunke N., Mehner T. (2003). Phosphorus uptake by *Microcystis* during passage through fish guts. Limnol. Oceanogr..

[72] Pollux B., Jong M., Steegh A., Ouborg N., van Groenendael J., Klaassen M. (2006). The effect of seed morphology on the potential dispersal of aquatic macrophytes by the Common Carp (*Cyprinus carpio*). Freshw. Biol..

[73] Agami M., Waisel Y. (1988). The role of fish in distribution and germination of seeds of the submerged macrophytes *Najas marina* L. and *Ruppia maritima* L.. Oecologia.

[74] Anderson J.T., Saldaña Rojas J., Flecker A.S. (2009). High-quality seed dispersal by fruit-eating fishes in Amazonian floodplain habitats. Oecologia.

[75] Jančula D., Míkovcová M., Adámek Z., Maršálek B. (2008). Changes in the photosynthetic activity of *Microcystis* colonies after gut passage through *Nile tilapia* (*Oreochromis niloticus*) and silver carp (*Hypophthalmichthys molitrix*). Aquac. Res..

[76] Lopes V.M., Costa P.R., Rosa R., Duarte B., Caçador I. (2019). Effects of Harmful Algal Bloom Toxin on Marine Organisms. Ecotoxicology of Marine Organisms.

[77] Solino L., Reis Costa P. (2018). Differential toxin profiles of ciguatoxins in marine organisms: Chemistry, fate and global distribution. Toxicon.

[78] Tsukamoto K. (2009). Oceanic migration and spawning of anguillid eels. J. Fish Biol..

[79] Tsukamoto K., Aoyama J., Miller M.J. (2002). Migration, speciation, and the evolution of diadromy in anguillid eels. Can. J. Fish. Aquat. Sci..

[80] Bardonnet A., Riera P. (2005). Feeding of glass eels (*Anguilla anguilla*) in the course of their estuarine migration: New insights from stable isotope analysis. Estuar. Coast. Shelf Sci..

[81] Chrisafi E., Kaspiris P., Katselis G. (2007). Feeding habits of sand smelt (*Atherina boyeri*, Risso 1810) in Trichonis Lake (Western Greece). J. Appl. Ichthyol..

[82] Harrison P.J., Waters R.E., Taylor F.J.R. (1980). A broad-spectrum artificial seawater medium for coastal and open ocean phytoplankton. J. Phycol..

[83] Fagín E., Bravo I., Garrido J.L., Rodríguez F., Figueroa R.I. (2019). *Scrippsiella acuminata* versus *Scrippsiella ramonii*: A Physiological Comparison. Cytometry A.

[84] R Core Team (2020). R: A Language and Environment for Statistical Computing.

